# Supplementary material for: A GNN-based approach for accurate trade balance forecasting and interpretable analysis
Source: PLoS One. 2026 Apr 29;21(4):e0346324. doi: 10.1371/journal.pone.0346324 (PMC13127960; doi:10.1371/journal.pone.0346324)
Supplement: Attachment — Submitted filename: plos_one_authorResponse.docx [file pone.0346324.s001.docx]

Review Comments to the Author

Please use the space provided to explain your answers to the questions above. You may also include additional comments for the author, including concerns about dual publication, research ethics, or publication ethics. (Please upload your review as an attachment if it exceeds 20,000 characters)

Reviewer #1: The author presented very interesting study in the domain of "A Random Forest Regression-based Approach for Accurate Trade Balance Forecasting" so i recommend Major revision before the final approval of the manuscript.
Concern 1) ROC and AUC are classification metrics, not regression metrics. Using them for regression (Section 5.6) is methodologically incorrect and shows conceptual misunderstanding. Kindly revise and correct it

Author Action: We have addressed this issue and corrected the conceptual misunderstanding. As we have incorporated GNN to find the complex relation between trade values, we added the metrics that are related to the forecast regression values, such as MSE, RMSE, MAE, and R-squared value. The R-squared analysis can be found in section 5.4, line numbers 583-610.
Concern 2) The model explains only 25% of variance too low to claim accurate forecasting. Conclusions overstate model performance.

Author Action: This valuable concern is addressed, and after realizing this mistake, we have replaced our model with GNN, and GNN is highly applicable to find the most complex relation between features. The GNN shows the R-squared value is 0.91, and shows accurate forecasting. The illustration of the R-squared value is shown in sections 5.1 and 5.2, line numbers 537-567.
Concern 3) The manuscript alternates between predicting “trade balance” and “export value.” Section 4.3 defines the target as export value, contradicting the abstract and other sections.

Author action: We have corrected the inconsistency of predicting value. We have forecasted the trade value, not the export value. We have changed the word where this inconsistency showed up.
Concern 4) Despite poor R², the study repeatedly claims Random Forest “outperforms” deep models and provides “accurate predictions.

Author Action: We understand the poor performance; we were not able to find the complex pattern with the trade values, so we employed GNN based approach, and this shows accurate forecasting results. GNN shows the R-squared value is 0.91, and shows accurate forecasting. The illustration of the R-squared value is shown in sections 5.2 and 5.3, line numbers 554-582.
Concern 5) There’s no significance analysis for performance differences between models. Kindly revise and correct it.

Author action: To address this concern, we have evaluated the different model performances using MSE, RMSE, MAE, and R-squared value. This can be found in section 5.4. Also, statistical significance analysis using the t-test can be found in section 5.6.1. and the Wilcoxon signed rank test that can be found in section 5.6.2.
Concern 6) Add an acronyms section before the references.

Author action: This concern is addressed, and we have added the acronyms before the references.
Concern 7) Motivation behind the study is missing in the manuscript.

Author action: To address this concern, the introduction section is modified so that the motivation of this study is clear to the readers.
Concern 8) Negative R² values for DNN, Transformer, and Ensemble models (Table 1) indicate poor model fitting. Such results should trigger re-evaluation, not comparison for superiority.

Author action: We have re-evaluated these performances; these values are negative because the trade values are in a very high range, and this model failed to find that pattern, showing a huge difference between the actual and predicted values. To validate this analysis, we used statistical methods in section 5.6. We evaluated two testing methods in section 5.6.1 and in section 5.6.2. In both of the test the statistics were poor and suggest that these models are not significant. Conversely, the GNN method shows significant and reliable.

Decision: Major Revision

Reviewer #2: This manuscript presents a comparative study of machine learning models for forecasting global trade balances. The topic is relevant, and the scale of the study (229 countries) is a notable contribution. However, the manuscript requires significant revisions to strengthen its methodological rigor, contextualization within the existing literature, and the depth of its analysis and discussion before it can be considered for publication.

Concern 1) Lack of Methodological and Literature Context:
Methodological Rationale: The manuscript would be significantly strengthened by including a dedicated section that justifies the selection of the specific machine learning models (e.g., DNN, Transformer, ensemble, and Random Forest). The authors should explain the anticipated strengths of each model in the context of trade data characteristics (e.g., non-linearity, high dimensionality, temporal aspects).
Critique of Previous ML Applications: The introduction and literature review should add more critical synthesis of previous studies that used ML for trade forecasting. Specifically, the authors should clearly delineate the common methodological limitations in prior work (e.g., over-reliance on single models, inadequate feature engineering, lack of interpretability) to better position their own research contributions

Author action: To address this valuable concern, we have given a dedicated section that justifies the selection of the specific machine learning models in section 5.8, line numbers 724-757. Furthermore, we have modified the introduction and literature review in sections 1 and 2 to add more critical synthesis of previous studies that used ML for trade forecasting and described the methodological limitations of prior studies.

Concern 2) Inadequate Description of Experimental Setup:
Data Partitioning and Validation: The manuscript misses a critical description of the data splitting strategy (e.g., train/validation/test sets) and the validation protocol. It is essential to detail whether a temporal split was used to preserve the time-series nature of the data and to explicitly state the measures taken to prevent data leakage, as this is fundamental to the credibility of the results.

Author Action: We have added the data partitioning and validation protocol, and adjust it in section 3.1, line numbers 204-217.

Concern 3) Lack of Robustness and Interpretability Analysis:
Sensitivity Analysis: To validate the robustness of the findings, the authors should perform sensitivity analyses; like:
Geographical Robustness: Re-running the analysis on economically distinct subgroups (e.g., OECD vs. non-OECD nations).
Temporal Robustness: Evaluating model performance on different time periods to test for temporal consistency.
Methodological Robustness: Repeating the feature selection process with alternative methods to ensure the stability of the selected features.

Author Action: T0 address this issue, we added a section in 5.7 (Sensitivity Analysis), where we analyzed the Geographical Robustness, Temporal Robustness, and Methodological Robustness.

Concern 4) Model Interpretability: The value of the study for policymakers and economists would be greatly enhanced by incorporating model interpretation techniques. Employing SHAP (Shapley Additive exPlanations) analysis or presenting normalized variable importance plots would help answer critical questions about which economic indicators are most influential globally and the direction of their impact on trade balances.

Author Action: The model interpretability with SHAP analysis can be found in section 5.9, where we analyzed SHAP-Based Feature Importance, Comparison with Alternative Importance Measures, AND SHAP visualization.

Concern 5) Insufficient Discussion and Acknowledgment of Limitations:
Discussion Section: The manuscript is currently missing a dedicated discussion section. This section should interpret the results in the context of the research questions and the existing literature. The striking performance gap between Random Forest and all other models (point #6) requires particularly careful interpretation.

Author Action: We added the discussion section 6, and discussed the limitation in section 7, line numbers 822-833.

Concern 6) Study Limitations: A balanced academic paper must acknowledge its limitations. The authors should discuss:
* The modest explanatory power (R² = 0.254) of the best model, indicating significant unexplained variance.
* The potential reasons for the catastrophic failure of complex models like DNN and Transformer.
* Challenges associated with data heterogeneity and imputation across 229 countries.
* The limitations of a static modeling approach that may not account for temporal dependencies and structural breaks.

Author Action: We have added the limitation of this study in in section 7, line numbers 822-833.
Concern 7) Results Presentation and Clarity:
Table 1 & 2: The results show that all models except Random Forest perform catastrophically poorly (with extreme negative R² values). The authors must explicitly address this in the text, explaining that these models failed to outperform a simple mean baseline and discussing the potential reasons for such a significant performance disparity. Presenting this as a standard model comparison is misleading.

Author Action: We have re-evaluated these performances; these values are negative because the trade values are in a very high range, and this model failed to find that pattern, showing a huge difference between the actual and predicted values. To validate this analysis, we used statistical methods in Section 5.6. We evaluated two testing methods in section 5.6.1 and in section 5.6.2. In both of the test the statistics were poor and suggest that these models are not significant. Conversely, the GNN method shows significant and reliable.

Concern 8)
Figure 4: The x-axis labels are currently overlapping, obstructing visual understanding. This figure should be reformatted for clarity.

Author action: We addressed this concern and modified the figure with no overlap.

Concern 10)
Figure 5: The caption for Figure 5 should be clarified. It is currently unclear what the "country feature" represents—does it indicate results for a specific country, or a specific feature related to countries?

Author Action: We have modified the caption and also described it in the description of this figure.

**Recommendation**
**Major Revision.** The manuscript has the potential to be a valuable contribution but requires substantial revisions to address the major concerns outlined above, particularly regarding methodological justification, validation, interpretability, and the discussion of results and limitations.
