# Supplementary material for: A GNN-based approach for accurate trade balance forecasting and interpretable analysis
Source: PLoS One. 2026 Apr 29;21(4):e0346324. doi: 10.1371/journal.pone.0346324 (PMC13127960; doi:10.1371/journal.pone.0346324)
Supplement: Attachment — Submitted filename: Responses to Review Comments_Liu.docx [file pone.0346324.s002.docx]

**Responses to Review Comments**

Thank you for giving us the opportunity to submit a revised version of our manuscript. We greatly appreciate the time and effort that you and the reviewers have dedicated to providing insightful and constructive feedback.

We have carefully considered all comments and revised the manuscript accordingly to meet PLOS ONE’s publication criteria. Below, we provide a point-by-point response to the reviewers' comments and the editorial requirements.

**Part I: Reply to Editorial Requirements**

1. **Code and Data Availability:** To fully comply with PLOS ONE's data and code sharing policies, we have updated our Data Availability Statement in the submission system.

- Data Availability: The raw trade statistics data underlying the findings of this study are publicly available from the International Trade Centre (ITC) TradeMap database and can be accessed at: .
- Code Availability: All author-generated code underpinning the findings in this manuscript is fully provided as a Supplementary Material file and will be published alongside the article without restrictions.

2. **Formatting and File Types:** We have ensured that the revised manuscript, title, and affiliations strictly follow PLOS ONE's style templates. We have uploaded the clean compiled PDF ("Manuscript"), the PDF with highlighted changes ("Revised Manuscript with Track Changes"), the LaTeX source files (".tex" as "Other"), and the Supplementary Material files ("Supporting Information") according to the journal's specific guidelines.

**Part II: Reply to Reviewer #1**

**Comment:** *I accept the article as authors addressed my all the comments carefully, no further comments from my side.*

**Response:** We sincerely thank the reviewer for recognizing our efforts and for the positive evaluation of our work. Your earlier feedback was instrumental in improving the overall quality and rigor of this manuscript.

**Part III: Reply to Reviewer #2**

**Comment 1:** *Typographical Error: There is a spelling error in Figure 1. Please review the figure caption or the text within the figure for the indicated misspelling and correct it.*

**Response:** We thank the reviewer for catching this oversight. We have corrected the spelling error in the flowchart of Figure 1 (changed "Explatory Data Analysis" to "Exploratory Data Analysis") and updated the high-resolution image in the revised manuscript.

**Fig 1.** Proposed Methodology

**Comment 2:** *Figure Readability: In Figure 3, the text/levels appear to be overlapping, which makes them difficult to read. Please adjust the layout, font size, or spacing to ensure all elements are clearly legible.*

**Response:** We entirely agree with the reviewer. We have replotted Figure 3 by introducing line breaks and adjusting the plot layout margins. The overlapping issue has been completely resolved, ensuring that all text, axis labels, and levels are now clearly legible without any truncation.

**Fig. 3** Exploring Relationships Between Export Value and Key Features

**Comment 3:** *Manuscript Length / Supplementary Material: The main text currently contains a high density of plots and tables. To improve the flow for the reader, I recommend moving some of the less critical plots or supplementary analyses to the Supplementary Materials section, keeping only the most essential figures in the main body.*

**Response:** We appreciate this excellent suggestion for improving the manuscript's flow and readability. We have significantly streamlined the main text by moving the auxiliary exploratory data plots and intermediate feature importance tables to the newly created Supplementary Materials file. Specifically, we have moved the "Average export value trends" bar plot (former Figure 4), the "Random Forest Importance" table (former Table 6), and the "Permutation Importance" table (former Table 7). These are now referenced in the main text as **S1 Fig**, **S1 Table**, and **S2 Table**, respectively. The main text now retains only the most essential figures and tables that directly support our key findings.

*“*$\cdots$ *S1 Fig, the first bar plot examines the average ’Value exported’ for different 349 ranges of the annual growth rate.* $\cdots$*”*

*“*$\cdots$ *S1 Fig ffers a clear, aggregated view of how the average ‘Value exported in 2023 (USD thousand)’ is influenced by these three key features.*$\cdots$*”*

*“*$\cdots$ *To validate the robustness of the SHAP-based interpretation, feature importance is further examined using Random Forest and permutation-based importance methods. S1 Table shows that historical trade balance values and global export share consistently rank among the most influential features, aligning closely with the SHAP results. Similarly, permutation importance (S2 Table) confirms the strong impact of squared balance and recent export values, reinforcing the reliability of the interpretability findings.* $\cdots$*”*

*“A GNN-based Approach for Accurate Trade Balance Forecasting and Interpretable Analysis*

**S1 Fig 1.** Average export value trends across feature levels.	This figure illustrates how average export values vary with key features in the dataset, including annual growth in value, share in world exports, and concentration of importing countries. Error bars provide a measure of the variability within each category.

**S1 TABLE 1.** Top Features Based on Random Forest Importance.

| **Feature** | **Importance** |
| --- | --- |
| Trade balance in 2022 (USD thousand) | 0.1538 |
| Trade balance in 2021 (USD thousand) | 0.1287 |
| Trade balance in 2020 (USD thousand) | 0.1246 |
| Share in world exports (%) | 0.1170 |
| balance 2022 squared | 0.1156 |
| Trade balance in 2019 (USD thousand) | 0.1096 |
| Value exported in 2019 (USD thousand) | 0.0613 |
| export 2022 squared | 0.0413 |
| Value exported in 2020 (USD thousand) | 0.0353 |
| Value exported in 2022 (USD thousand) | 0.0326 |

**S2 TABLE 2.** Top Features Based on Permutation Importance.

| **Feature** | **Importance** |
| --- | --- |
| balance_2022_squared | 0.0754 |
| Value exported in 2021 (USD thousand) | 0.0406 |
| Value exported in 2023 (USD thousand) | 0.0344 |
| Share in world exports (%) | 0.0315 |
| Value exported in 2019 (USD thousand) | 0.0254 |
| Trade balance in 2019 (USD thousand) | 0.0168 |
| Value exported in 2020 (USD thousand) | 0.0129 |
| Trade balance in 2021 (USD thousand) | 0.0112 |
| Value exported in 2022 (USD thousand) | 0.0109 |
| export_2022_squared | 0.0095 |

*”*

**Comment 4:** *Strengthening the Discussion: The final paragraph of the Discussion section currently makes a broad claim about the model's potential for policy analysis. To strengthen this, the authors should support their findings by citing specific references. For example, rather than stating that the findings are "consistent with emerging literature," please cite 1-2 specific papers that have similarly applied graph-based methods to economic or trade forecasting problems.*

**Response:** We thank the reviewer for this constructive feedback. We have revised the final paragraph of the Discussion section to firmly ground our claims in existing literature. We have explicitly cited two recent, highly relevant studies that successfully apply Graph Neural Networks to international trade networks and bilateral trade modeling. The revised sentence now reads:

*“*$\cdots$ *These findings are consistent with emerging literature that successfully applies graph-based methods to economic and trade forecasting [42, 43], and suggest that graph-based learning offers a meaningful advancement for trade forecasting applications, with strong potential for real-world policy analysis and decision-support systems.”*

Thank you once again for your constructive feedback and guidance. We believe these revisions have significantly strengthened our manuscript, and we hope the revised version is now fully suitable for publication in PLOS ONE.

Sincerely, **Cheng Ding** On behalf of all co-authors.

**Reference**

[42] Sellami B, Ounoughi C, Kalvet T,Tiits M, Rincon-Yanez D. Harnessing Graph 965 Neural Networks to Predict International Trade Flows. Big Data and Cognitive 966 Computing. 2024;8(6):65. doi:10.3390/bdcc8060065. 967.

[43] Panford-Quainoo K, Bose AJ.Bilateral Trade Modeling with Graph Neural 968 Networks; 2020.Available from:969 https://api.semanticscholar.org/CorpusID:218626926.
